# A randomised feasibility trial of an intervention to support sharing of HIV status for 18–25-year olds living with perinatally acquired HIV compared with standard care: HIV Empowering Adults’ Decisions to Share—UK/Uganda Project (HEADS-UP)

**DOI:** 10.1186/s40814-020-00688-w

**Published:** 2020-09-24

**Authors:** Michael Evangeli, Caroline Foster, Victor Musiime, Sarah Fidler, Janet Seeley, Georgina Gnan

**Affiliations:** 1 Royal Holloway University of London, Egham, Surrey, UK; 2 Imperial College Healthcare NHS Trust, London, UK; 3 Makerere University, Kampala, Uganda; 4 Joint Clinical Research Centre, Lubowa, Uganda; 5 Imperial College London, Department of Infectious Disease, London, UK; 6 Imperial College NIHR BRC, London, UK; 7 London School of Hygiene and Tropical Medicine, London, UK

**Keywords:** HIV, Disclosure, Perinatal, Feasibility, Intervention

## Abstract

**Abstract:**

**Background:**

Young adults with perinatally acquired HIV (PAH) face several challenges, including adhering to antiretroviral therapy (ART), managing the risk of onward HIV transmission and maintaining positive well-being. Sharing one’s HIV status with others (onward HIV disclosure) may assist with these challenges by facilitating emotional and practical support. Rates of HIV status sharing are, however, low in this population. There are no existing interventions focused on sharing one’s HIV status for young adults living with PAH. The HEADS-UP study is designed to develop and test the feasibility of an intervention to help the sharing of HIV status for young adults with PAH.

**Methods:**

The study is a 30-month multi-site randomised feasibility study across both a high-income/low-HIV prevalence country (UK) and a low-income/high-HIV prevalence country (Uganda). Phase 1 (12 months) will involve developing the intervention using qualitative interviews with 20 young people living with PAH (ten in the UK—18 to 29 years; ten in Uganda—18 to 25 years), 20 of their social network (friends, family, sexual partners as defined by the young person; ten in the UK, ten in Uganda) and ten professionals with experience working with young adults with PAH (five in the UK, five in Uganda). Phase 2 (18 months) involves conducting a randomised feasibility parallel group trial of the intervention alongside current standard of care condition in each country (main study) with 18- to 25-year olds with PAH. A sample size of 94 participants per condition (intervention or standard of care; 188 participants in total: 47 in each condition in each country) with data at both the baseline and 6-month follow-up time points, across UK and Ugandan sites will be recruited. Participants in the intervention condition will also complete measures immediately post-intervention. Face-to-face interviews will be conducted with ten participants in both countries immediately post-intervention and at 6-month follow-up (sub-study).

**Discussion:**

This study will be the first trial that we are aware of to address important gaps in understanding acceptable and feasible ways of delivering HIV status sharing support for young people living with PAH.

**Trial registration:**

ISRCTN Registry, ISRCTN31852047, Registered on 21 January, 2019. Study sponsor: Royal Holloway University of London. Sponsor contact: alicen.nickson@rhul.ac.uk. Date and version: April 2020. Protocol version 3.5.

## Background

The World Health Organisation estimates that globally, around 5 million young people (15–25 years) are living with HIV, and a significant proportion of these have acquired HIV perinatally (perinatally acquired HIV (PAH)) [1]. In some low-income countries, young people are disproportionately affected by HIV. For example, in Uganda, youth (aged 10–24) comprise 33% of the population, but account for nearly 50% of the people living with HIV in the country [2]. About 78% of young people aged 15–24 living with HIV reside in sub-Saharan Africa [3].

The enhanced availability of antiretroviral therapy (ART), as well as the change in ART guidelines since 2015 [4], recommending ART for all people living with HIV irrespective of CD4 count or clinical stage, has led to a growing proportion of perinatally HIV-infected children surviving into adulthood [5]. The UK has a relatively small number of people living with PAH compared with other countries globally [5]. The majority of the UK cohort is of sub-Saharan African origin, with half born outside the UK [6]. Uganda has a much larger number of people living with PAH, although there are no precise estimates of the size of this population [7].

Although young people living with PAH share some similar challenges to those who are behaviourally infected (e.g., potentially compromised health and risk of onward transmission through sex) other stressors relate specifically to perinatal transmission. Longstanding HIV infection acquired prior to physiological and immunological development results in distinctive chronic clinical complications that can cause severe morbidity [8]. In addition to dealing with chronic illness and its associated stressors (e.g., hospitalisations, missed school and social opportunities, and pain), young people living with PAH face specific unique additional difficulties around ART adherence and well-being, stemming from multiple caretaking transitions and loss due to parental illness or death, loss of siblings, stigma and discrimination, confrontation with mortality and an uncertain future [8].

The current generation of young adults with PAH have often additionally experienced suboptimal ART regimens, many being born in the era of mono- and dual-antiretroviral therapy, thus increasing their likelihood of drug resistance [9]. Rates of viral suppression are variable globally, associated with poor ART adherence [10]. Adherence to ART remains unsatisfactory and varies between 27% and 80% across different populations in various studies [11]. A systematic review of 50 studies from 53 countries reported a pooled level of 62% good adherence (mostly assessed in relation to viral suppression) among youth [10]. There is evidence of higher levels of adherence in some recent studies. For example, of young adults living with PAH who attended a tertiary youth-friendly service in London, UK, 81% had an undetectable viral load (< 200 copies RNA/ml) [12]. Despite improvements in viral suppression rates across specific subpopulations, the lowest rates of viral suppression are among young people aged 13–24 when compared with younger children or older adults [9]. A recent UK cohort study found that adolescents with HIV had poorer outcomes on ART (viral suppression and good immune status) compared with younger children [13]. This trend of poor outcomes has been observed to continue in young adults with perinatal HIV following transition to adult care, and data show lower rates of ART uptake in young adults living with PAH compared with youth with behavioural acquisition [14, 15] highlighting a key population who may need additional support to achieve the same level of treatment success [13].

ART adherence is dependent on retention in care. There are particular challenges in eastern and southern Africa to retaining people living with HIV in care, with a large number lost to follow up after initiating treatment, particularly for young people [16]. In this region, the proportion of all people with HIV who knew their status was 85% in 2018 with an estimated 67% on treatment, and 58% virally suppressed (up from 43% in 2015). There is a shortage of evidence on the most effective approaches for young people living with PAH failing to achieve or sustain viral suppression due to non-adherence [17].

Unprotected sexual intercourse in young people living with HIV in the context of globally variable ART coverage and low rates of viral suppression [18] presents a risk of onward (potentially drug resistant) HIV transmission to partners and to offspring. For many young adults, regardless of their HIV status, negotiating their first sexual experiences can be complex and the importance of condom use may vary depending on cultural and religious norms [19, 20]. For young people with PAH, sexual onset will occur alongside their knowledge of having a sexually transmittable, stigmatised medical condition.

Sharing an HIV-positive status with others has the potential to facilitate positive outcomes in the above areas. It is now accepted that successful viral suppression (< 200 copies HIV RNA/ml) with ART prevents HIV transmission to sexual partners (Undetectable = Untransmittable; U = U) [21, 22]. In situations where viral suppression has not been achieved, however, sharing an HIV-positive status with partners may reduce onward HIV transmission, by fostering communication about safer sex. Indeed, sharing HIV status with partners has been estimated to reduce risks of onward HIV transmission by 18–61% [23, 24]. HIV status sharing may also encourage a partner to undergo testing, use prevention strategies such as condoms, pre-exposure prophylaxis (PrEP) and post-exposure prophylaxis (PEP), and engage in care/treatment if needed [25].

Although young adults who have an undetectable viral load may not have to share their status to prevent onward HIV transmission, sharing has been shown to have a number of personal benefits in addition to its public health benefits [26]. Sharing may help to buffer HIV-related stress and improve well-being [27, 28]. Being open about one’s HIV status may facilitate obtaining social support from significant others such as families and peers, which in turn is a prerequisite for constructive coping, enhanced self-esteem and other health-promoting behaviors [28]. Sharing of one’s HIV status may also enhance ART adherence, due to greater adherence support from partners, friends or family and a reduced need to hide medication use in situations where sharing has not occurred [29, 30]. This is particularly important even for young adults who have an undetectable viral load as they will only remain undetectable if they continue to adhere to ART. Fear of sharing HIV status is commonly cited as a barrier to ART adherence in young people living with PAH [31]. There is also evidence of lower levels of HIV status sharing being associated with poorer engagement with HIV care [32, 33].

Globally, there are low rates of sharing an HIV-positive status with a partner, ranging from 39–97% [34]. This may be even lower in young people with PAH. Birungi, Obare, Mugisha, Evelia and Nyombi [35] reported that only 38% of their sample of youth with PAH in relationships (aged 15–19 years) had shared their HIV status with their current partner. Lee and Oberdorfer [36] found that 48% of their sample of adolescents (aged 13+) living with PAH had never shared their status to anyone. Only 40% of young adults living with PAH in the US reported sharing with all or most of their partners in a recent study, and almost half (45%) reported sharing with no partners when having unprotected sex [25]. In a study in Uganda and Kenya, only one in five participants (adolescents aged 13–17) reported having shared their HIV status with their peers [28].

Sharing an HIV status carries a unique challenge for young people with PAH with concerns about revealing their mother’s (and other family members’) HIV status [37]. Negative parental sharing attitudes, including directives to not share, may be internalised [37] with an atmosphere of secrecy and limits to open communication about HIV affecting the young person at home, in their community and in the clinic [38, 39]. The subjective difficulty of sharing one’s HIV status in young people with PAH, particularly in relationships, has been frequently reported, with a fear of rejection, a lack of confidence about sharing (disclosure self-efficacy), and fear of secondary disclosure from the recipient to others cited as barriers to sharing [37, 40–42].

There remain important risks of sharing ones’ HIV status, including the threat of rejection, humiliation, stigma and even violence [25, 43]. However, overall, there may be more potential benefits of sharing ones’ HIV status. Hence, for other groups of people living with HIV, there have been efforts to develop HIV status-sharing interventions. Conserve et al. [34] reviewed interventions promoting HIV sharing to sexual partners. Three of the five studies included in this review (four of which focused on MSM, the other minority women) showed rates or frequencies of HIV sharing that were greater in the intervention condition than the control condition, with small-to-medium effect sizes reported. Kennedy et al. [44] reviewed interventions promoting HIV status sharing to *any* recipient in low- and middle-income countries. Seven of the nine studies where outcomes were reported showed a significantly greater level of HIV sharing in the intervention compared with the control arm, with small-to-medium effects. There are no HIV status-sharing interventions specifically designed for young people with PAH, or young people with HIV more generally [45]. Facilitating sharing is often a small component of multi-component interventions for youth with HIV, but this component is rarely evaluated [46]. A recent systematic review of interventions to improve retention in HIV care and adherence to ART among youth highlighted the need for further development and testing of multi-faceted interventions, to address broader social barriers to adherence and retention [47].

Consistent with the gaps in the evidence base outlined above, there is a lack of sharing guidance to support young people with PAH or professionals working with this population [45]. The World Health Organisation (WHO) has called for work in this area, specifying the need for interventions to help adolescent disclosure decision-making, support caregivers and train providers (WHO, 2018). There is evidence that young people with HIV [48–50] and health care workers [51] would like more HIV status-sharing support.

Given the gap in the evidence base, and the importance of the issue, we aim to develop and test the feasibility of a behavioural intervention to increase sharing levels and satisfaction with sharing decisions in perinatally infected young adults in the UK and Uganda in a 30-month project. The appropriateness of the intervention in high-income/low-prevalence (e.g., UK) and low-income/high-prevalence (e.g., Uganda) contexts needs to be assessed, given evidence of low rates of sharing in both. The target population will be aged 18–25 years, due to higher rates of sexual activity, with potentially more active consideration of sharing than in younger populations, as well as higher mortality and morbidity in this age group [12]. In addition, in this age range, decisions about sharing may be less constrained by others than during earlier adolescence. The focus will be on sharing with any recipient depending on participant preference. Enhancing sharing with one category of recipient could facilitate sharing with other categories. For example, increased sharing and communication with friends and family has been shown to be associated with sharing with a partner [25, 42].

We hypothesise that (1) the intervention will be feasible, in relation to recruitment, retention and acceptability and (2) participants in the intervention group will have a higher rate of sharing in the previous 6 months at follow-up than participants in a standard-of-care (SOC) group.

## Methods/design

### Study settings

The study will take place in two countries (list of study sites available on request):

1. UK. Participants will be recruited from five inner city NHS clinics which provide services for young people living with PAH across two cities, as well as from one UK-based HIV charity.
2. Uganda. Participants will be recruited from a not-for-profit organisation in one city, providing HIV care to young people living with PAH ≥ 18 years.

### Phase 1—intervention development and adaptation (12 months)

This phase will involve (1) assessing barriers and facilitators to HIV status sharing, with questions informed by an intervention development framework [52], (2) developing a theory of HIV sharing relevant to the study contexts, (3) developing intervention and recruitment strategies, (4) carrying out formative assessment of intervention components for feasibility, understanding and acceptability, (5) manualising the intervention, (6) developing semi-structured interview guides, (7) developing a fidelity measure for the intervention, (8) developing a measure to describe standard of care, (9) training therapists delivering the intervention, (10) developing methods to assess intervention costs in phase 2 and (11) developing and adapting measures of primary and secondary outcomes and other variables (e.g., ART adherence self-efficacy). The latter task will involve producing linguistically and culturally validated measures where none exist for the Ugandan context, through translating existing questionnaires/items and response options, back translation by a different translator, reviewing of translated items by a local expert panel and cognitive interviewing of ten young people taking part in phase 1 in Uganda.

Individual assessments and focus groups will be used. Participants for this stage will involve the following:

- Ten young people with PAH in the UK and ten in Uganda:
  - *Inclusion criteria:*
    - Aged 18 to 29 years inclusive in the UK, and 18 to 25 inclusive in Uganda (the wider age range in the UK reflects the greater difficulty in recruiting from the population, given its relatively smaller size compared with Uganda);
    - Living with PAH;
    - Knowledge of own HIV status;
    - Able to give informed consent
  - *Exclusion criteria*:
    - Current serious mental health problems. Clinician opinion will be sought regarding the assessment of any mental health difficulties that might render young people unsuitable for the study;
    - Moderate-to-severe learning disability/executive functioning difficulties. Individuals with clinically documented moderate-to-severe cognitive difficulties will be excluded;
    - Current serious physical health problems with life expectancy < 12 months, according to clinician opinion;
    - Current participation in other psychosocial intervention/support research
- Friends, family and partners of young people with PAH in the UK (ten individuals) and in Uganda (a further ten).
  - *Inclusion criteria*:
    - Friends, family or partners of people currently or recently aged 18–29 years living with PAH in the UK, 18–25 years in Uganda;
    - Awareness of the HIV status of the young person with PAH;
- Five professionals working with young people living with PAH in the UK and five in Uganda
  - *Inclusion criteria*:
    - Current or recent involvement in the care of the people aged 18-25 living with PAH.

### Phase 2—main study: feasibility trial

#### Study design

**Fig. 1:**
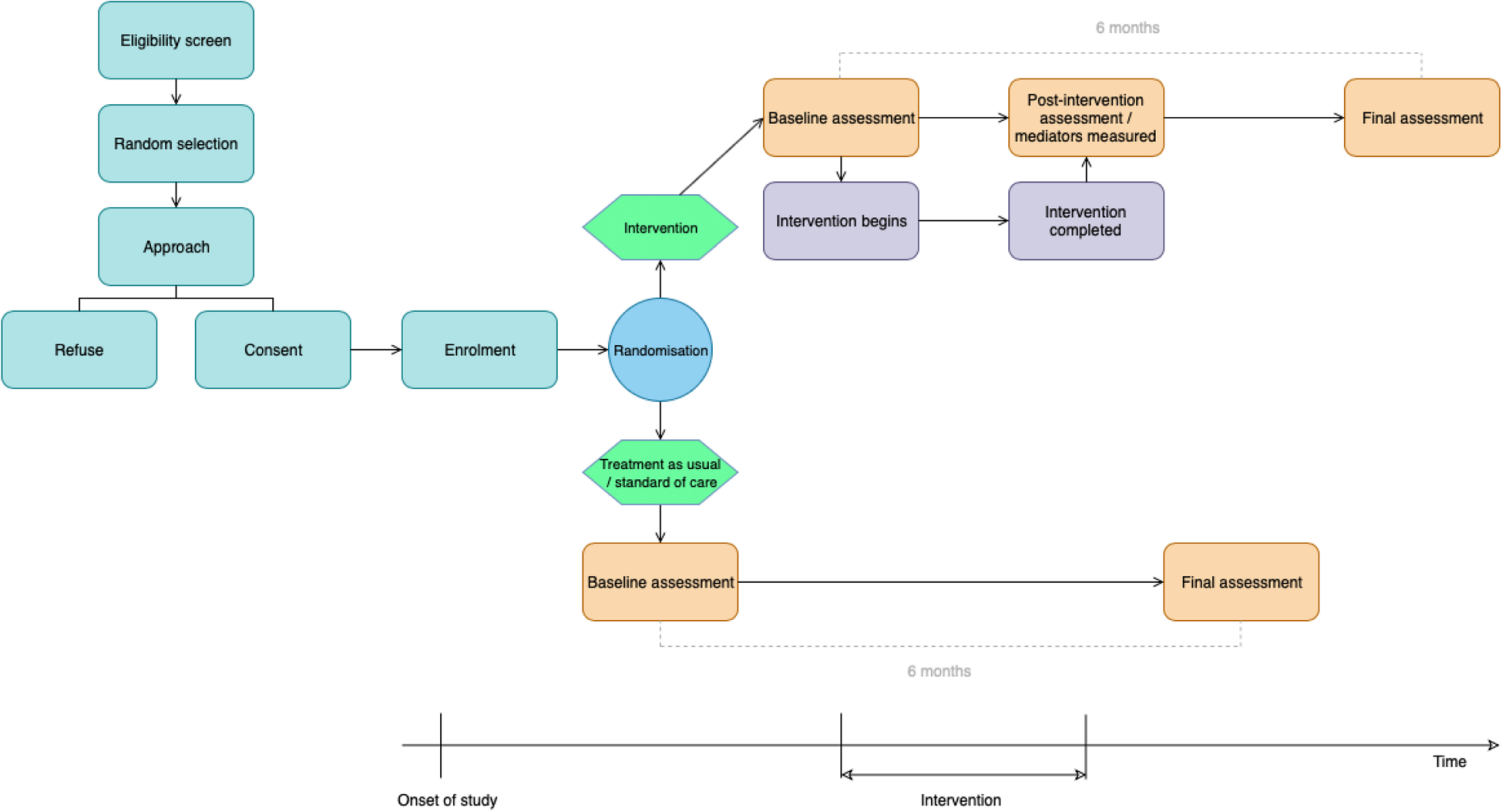
Flow diagram of Phase 2 study design

This component of the study uses a multi-site randomised feasibility design. Participants will be randomised to either the intervention or standard of care. Assessments will be carried out at three time points—pre-intervention/baseline (for both conditions), post-intervention (at the end of the final session, only for the intervention condition), and 6-month follow-up (6 months from baseline, both conditions). See Fig. 1 for a flow diagram of the Phase 2 study design.

### Sample

The sample size will be 94 per condition (intervention or standard of care; 188 participants in total: 47 in each condition in each country). This sample size has been chosen to allow for study attrition so that at least 64 participants per condition will be retained in the study.

#### Sample size calculation

The sample size of 32 in each condition in each country has been chosen to both assess primary outcomes, and to be sufficient to enable differences of between 0.5 and 1 standard deviation to be observed between conditions with 80% statistical power across secondary outcomes (e.g., changes in rates of HIV status sharing). Half a standardised difference would require 64 per condition, and one standard deviation difference would require 16 per condition.

#### Inclusion criteria

The following are included in the study:

- Aged 18 to 25 years inclusive;
- Living with PAH;
- Receiving HIV care at study sites;
- Knowledge of own HIV status

#### Exclusion criteria

The following are excluded in the study:

- Current serious mental health problems. Clinician opinion will be sought regarding the assessment of any mental health difficulties that might render young people unsuitable for the study;
- Moderate-to-severe learning disability/executive functioning difficulties. Individuals with clinically documented moderate-to-severe cognitive difficulties will be excluded.
- Current serious physical health problems with life expectancy of < 12 months, according to clinician opinion;
- Inability to understand or communicate in English (UK) or either English or Luganda (Uganda) according to clinician opinion
- Current participation in other psychosocial intervention/support research
- Participation in phase 1 of this study
- Participation of another individual within the household

### Intervention condition

The intervention condition will consist of four sessions (90 min per session) delivered in a mixed group/individual format (three group sessions including seven or eight participants/one individual session); professionally led (therapist one); mixed gender; face-to-face; with peer mentor involvement (therapist two) and follow-up support. These features were preferred by clinic attendees in survey of 57 young people with PAH in the UK [53] and were seen as feasible in a second survey of 16 professionals working with this population in the UK [54].

The intervention will aim to develop motivation and skills for HIV status sharing, increase HIV status sharing self-efficacy, decrease anxiety about HIV sharing and increase satisfaction with HIV status sharing decision-making. Motivational interviewing (MI) and Cognitive behavioural therapy (CBT) will guide the intervention. The intervention will be delivered by two therapists in each country. One will be a health professional (not a mental health specialist). The other will be a peer worker.

The content of the intervention is based on models of HIV disclosure decision-making and HIV disclosure anxiety [55–58] and existing evidence of HIV status-sharing correlates, barriers and facilitators [25, 28, 59]. In particular, disclosure attitudes, normative beliefs, self-efficacy, planning and support are argued to be important proximal determinants of HIV status sharing. These factors are suggested to be influenced by more distal factors: personal values and HIV stigma.

The content of specific sessions will be as follows:

- Session 1—Engaging: ground rules, living with HIV, previous experiences of HIV sharing (and evaluation of these experiences), defining HIV status sharing (as a process), understanding HIV status-sharing anxiety, risks and benefits of disclosure and non-disclosure in specific situations and with different recipients, and values clarification exercise
- Session 2—Focusing and evoking: HIV sharing wants and needs, eliciting and reinforcement of personal HIV-sharing decision rules, exploring discrepancy between decision rules/current HIV status sharing and values, and examining evidence for HIV status-sharing anxiety
- Session 3—Developing HIV status-sharing skills: identifying barriers and facilitators to HIV status sharing, responding to others’ reactions to HIV status sharing, HIV sharing strategies; developing HIV-sharing decision (if…then) rules, HIV status-sharing communication skills, use of social support (identifying safe/special people to disclose to), managing HIV status-sharing anxiety (challenging anxious thinking), HIV-sharing hierarchies including level of HIV sharing and level of anxiety about response, setting HIV-sharing goals, and planning to disclose (how, where and when)
- Session 4—Individual goal setting and planning: personal feedback from assessment; assessing HIV-sharing readiness; goal setting; who, how and when to disclose (action planning, using HIV-sharing decision aid); coping with HIV-sharing consequences over time (coping planning); using pre and post HIV-sharing support for self-disclosure recipient; safer sex skills (including condom use); considering nature of relationship with potential recipient, level of HIV sharing, and method of HIV sharing; ways to minimise risk of adverse outcomes/overcome barriers/respond to adverse responses; accepting possibility of adverse outcomes; and producing a personalised HIV-sharing plan

We will use role play, modelling, cognitive restructuring and behavioural exposure techniques, with both video material and group discussions integrated with interactive exercises. We will aim to space out the sessions to allow reflection on HIV sharing or actual sharing to take place between sessions. Follow-up emotional, informational and problem-solving support (in the 6 months from baseline to the follow-up data point) from a peer worker will be provided for both young people exposed to the intervention and people whom they have disclosed to. This will involve the use of both phone and online/social media options [60].

### Standard-of-care condition

The UK NHS sites and the Uganda site have dedicated clinics for young people growing up with HIV who have previously been in paediatric care, with peer support and professional psychosocial support available in the Uganda site and in the majority of the UK NHS sites. In these clinics, young people can discuss HIV sharing with their multidisciplinary team, attend with their partner, and have access to a range of other services (e.g., HIV testing, PrEP and condom provision). Standard of care in both countries, however, is for there to be no routine or structured psychosocial intervention to facilitate HIV sharing or sharing decision-making.

### Measures

Information on viral load and CD4 counts, as available, will be collected from clinic records. Scales in English will be translated into Luganda during the intervention development phase and adapted as necessary.

#### Primary outcome measure

**Study recruitment and retention**

The feasibility of the intervention will be assessed in relation to study recruitment, retention and acceptability. Recruitment and retention rates will be calculated for each site and for each condition. In addition, post-intervention rating scales will be administered to assess acceptability for participants in the intervention condition (satisfaction, intention to continue to use, and perceived appropriateness of the intervention) [61].

#### Secondary outcome measures

The range of outcomes reflect the likely diversity of the sample and the potential complexity of relationships between sharing, its predictors and consequences.

**HIV disclosure behaviour**

Self-reported HIV-sharing events will be assessed through recording the frequency of new disclosures (full or partial; first hand or second hand with consent) in the last 6 months to partners, friends and family, and any change in the proportion of social network disclosed to.

For each disclosure, recipient and relationship characteristics, perceived satisfaction with HIV-sharing decision, and the nature of the recipient’s response will be assessed. In addition, for those individuals in a participant’s social network not disclosed to, satisfaction with sharing decisions and disclosure intention will be measured. Any social harms occurring as a result of sharing will be recorded. We will develop this measure with reference to existing relevant measures [62–65]. At the follow-up time point for those in both conditions, we will also ask about whether any disclosure support was sought from professionals or social contacts during the previous 6 months.

**ART adherence**

Biological correlates of ART adherence (most recent viral load and CD4 count, if available) will be collected from participants’ clinical records. Adherence behaviour will be assessed by the CASE adherence index [66], which has been used in Uganda [28]. The CASE adherence index contains three questions relating to difficulty in taking ART medication on time (scored on a scale from 4—never to 1—all of the time), frequency of missed doses (scored on a scale from 1—everyday to 6—never) and time since most recent missed dose (scored on a scale from 1—within the past week to 6—never). Its reliability and validity has been assessed by estimating the degree of sensitivity and specificity to changes in three-day adherence self-report and comparing it to changes in HIV virologic outcomes and CD4 counts across time [66].

**Psychological well-being**

The 6-item psychological domain from the World Health Organisation Quality of Life brief questionnaire (WHOQOL BREF) [67] will be used. This measure has been translated into Luganda with good evidence of reliability and validity [68]. It includes questions on bodily image and appearance, negative feelings, positive feelings, self-esteem, spirituality/religion/personal beliefs, and thinking/concentration, which are answered on a 5-point scale (e.g. from 1—not at all to 5—completely). As well as measuring wellbeing as an outcome of the intervention, this variable may also mediate changes in other outcomes.

**Social support**

The 6-item Social Support Questionnaire Short form (SSQ6) [69] will be used. For each of the 6 items (e.g. Whom can you really count on to help you feel more relaxed when you are under pressure or tense?), respondents indicate the number of people available to provide support and then rate the overall level of satisfaction with the support given in each of the areas from 6—very satisfied to 1—very dissatisfied. A longer form of this measure has been used with young people living with HIV in Uganda [70]. As well as measuring social support as an outcome of the intervention, this variable may also mediate changes in other outcomes.

**Hope**

The 6-item State Hope Scale will be used [71]. The State Hope Scale offers a brief, internally consistent, and valid self-report measure of ongoing goal-directed thinking [71]. It includes items such as “There are lots of ways around any problem that I am facing now”, and is scored on an 8-point Likert scale from 1 (definitely true) to 8 (definitely false).

**Sexual outcomes**

The frequency and rate of unprotected sexual intercourse will be calculated for casual and regular partners with self-report data. Pregnancy, perceived partner status and PrEP use will also be recorded.

**Decisional conflict scale**

The 4-item decisional conflict scale will be used in relation to decisions to share [72]. The four items are the following: feeling uncertain (**S**ure of myself), feeling informed (**U**nderstand information), feeling clear about values (**R**isk-benefit ratio), and feeling supported in decision making (**E**ncouragement). A response of yes scores 1 and a response of no scores 0; a score of < 4 is a positive result for decisional conflict. The internal consistency of the scale has been shown to be moderate in English-speaking treatment-option patients [72], and the scale has shown adequate psychometric properties in a primary care population with a low prevalence of clinically significant decisional conflict [73].

#### Measurement of hypothesised mediators

**HIV disclosure cognitions and affect**

We will translate and adapt the 18-item HIV disclosure cognitions and affect scale, which has shown good preliminary evidence of reliability and validity in adolescents with PAH [74]. This scale contains three subscales representing negative disclosure attitudes and feelings, disclosure self-efficacy and positive disclosure attitudes and feelings. An example item is, “I am worried they will tell others”. Items are scored on a 5-point Likert scale from strongly disagree to strongly agree. We will develop an HIV disclosure intention item from existing measures [25, 75].

**HIV disclosure planning**

HIV disclosure planning will be assessed by asking two questions relating to action and coping planning. These questions will be adapted from questions used in the study with adolescents with PAH in the UK, from which a novel way of coding HIV status-sharing planning was developed [76].

**ART adherence self-efficacy**

The 14-item behavioural skills subscale of the Life Windows Adherence Questionnaire [77] will be used. This has shown good reliability in different contexts, including in sub-Saharan Africa [78–80]. An example item is, “How hard or easy is it for you to remember to take your HIV medications?”. Response options are 1 (cannot do at all) to 5 (certain you can do).

**HIV stigma**

The three-item negative self-image subscale from the short form of the HIV stigma scale will be used [81]. This has shown evidence of good reliability. An example item is “I feel I’m not as good a person as others because I have HIV”, and responses are on a 4-point Likert scale, ranging from strongly disagree (1) to strongly agree (4).

#### Measurement of background variables

**Previous HIV disclosure**

In addition to recording individual disclosure events in the previous 6 months (above), and the proportion of friends, family and partners disclosed to [63–65], participants will be asked about lifetime HIV-sharing frequency.

**Personal values**

The 24-item Agentic and Communal Value Scale (ACV) will be used [82]. The 24 items include 12 Agency items (e.g. wealth, pleasure, ambition) and 12 Communion items (e.g. forgiveness, trust, politeness) which are rated in terms of the relative importance of each value as “a guiding principle in my life” from not important to me (1) to highly important to me (9). The measure has been used with young adults [83, 84], including those with PAH [85] and has evidence of good reliability.

**Demographic and clinical variables**

Demographic variables (current age, ethnicity, gender, parental loss, relationship status, substance use, pregnancy, housing, location, living situation, education/occupation, income/socio-economic status, sexuality) and clinical variables (age at paediatric HIV disclosure, previous psychosocial interventions, clinic, clinic attendance, regimen, side effects, comorbid health conditions) will be assessed. We will record whether psychosocial support has been offered to the participants over the course of the study in both countries and the nature of any discussions about HIV sharing in standard care. This information will be obtained from participants (during the follow-up assessment) and clinic staff (using standard-of-care monitoring forms).

### Phase 2—Sub-study: qualitative interviews

Face-to-face interviews with ten participants in the UK and ten participants in Uganda will be conducted post-intervention (immediately after the final intervention session, and at the 6-month follow-up) to aid in the assessment of feasibility and to clarify potential causal mechanisms of the intervention. Individuals will be sampled purposively from both conditions (interventions and standard of care) to ensure that a range of participant characteristics (e.g. age, gender, level of HIV sharing) are represented.

#### Procedure

**Phase 1**

***Sampling and identification***

Young people with PAH meeting the inclusion criteria will be identified by clinicians and representatives of support organisations with an attempt to sample those with a range of levels of onward HIV disclosure and with a range of demographic characteristics. Friends, family and partners of young people with PAH will be identified by the young person with PAH. Professionals working with young people with PAH will be identified by the research team, attempting to sample across a range of clinics and support organisations.

***Approach and provision of study information***

Young people identified will be informed about the study verbally by a named clinician or support organisation representative by phone or at their next clinic/support organisation appointment. If they are informed by phone and are interested in the study, they will be asked permission for their professional to pass on their contact details to the study coordinator. The study coordinator will contact the young person to arrange a time to meet and to go through the participant information sheet. If the young person is informed at their next clinic/support organisation appointment, they will be given the participant information sheet by the professional who will either go through this with them or ask the study coordinator to do this, if they are available. If the young person is interested in taking part, having gone through the participant information sheet with the professional, their details will be passed on to the study coordinator.

Young people will be asked whether they can identify any friends, family members or regular partners who they think might like to take part in the study. The young person will be asked to discuss the project with them and to provide the study coordinator’s details. If friends, family or partners are interested in finding out more about the study, they will be asked to contact the study coordinator by phone. If they are interested, they will arrange a time to meet the study coordinator to go through the participant information sheet. For professionals working with young people with PAH, the study coordinator will inform them about the study by phone. If they are interested, they will arrange a time to meet the study coordinator to go through the participant information sheet.

***Consent and participation***

Written informed consent will be sought by the study coordinator or a member of the clinical team. Participants will be asked to offer their consent for all elements of this phase of the study, despite the fact that they may not be asked to take part in all activities. The right to withdraw will be stressed. One copy of the signed consent form will be given to the participant and one will be kept by the study coordinator to file in the central study register. The participant will be enrolled and then they will participate in the study. Travel expenses will be paid and participant payments will be made to non-professional participants and receipts will be kept by the study coordinator.

**Phase 2: main study**

***Sampling, identification, screening and randomisation***

A sampling frame will be constructed for each site by a named clinician applying the inclusion and exclusion criteria to their clinic population. A record of young people who are not eligible will be kept in a site study log to ensure that these individuals are not screened or approached in the future. Anonymised details of those not eligible (with reasons) will be passed to the study coordinator to record in the central study register to enable a study flow diagram to be compiled. The details of those eligible to participate will be provided to the study coordinator. Simple random sampling (without replacement) will be used by the study coordinator to identify which young people are approached to take part in the study from among those eligible across the sites in the country (using Research Randomizer: https://www.randomizer.org/).

***Approach and provision of study information***

If young people are eligible and randomly selected to be approached, the study coordinator will inform a named clinician at each site. The young person will be informed about the study verbally by the named clinician by phone, text or email, or at their next clinic appointment. If the young person expresses an interest in the study, they will be given further information by either the clinician or the study coordinator (the latter after permission for the clinician to pass on their contact details to the study coordinator has been sought). If the study coordinator is the person providing further information, the study coordinator will contact the young person to arrange a time to meet and to go through the participant information sheet. If the clinician is the person providing further information, the young person will be given the participant information sheet by the clinician who will go through this with them. If the young person is interested in taking part, having gone through the participant information sheet with the clinician, their details will be passed on to the study coordinator. If potential participants are not interested in taking part, this will be recorded in the site study log by the named clinician and anonymised information (with the reason for refusal) will be provided to the study coordinator to record in the central study register. This will help to determine the study response rate.

***Consent***

Written informed consent will be sought by the clinician or the study coordinator in each country. Consent documents will be translated into Luganda in Uganda, with the participants in Uganda having the choice between English or Luganda documents. Young people will be asked to offer their consent for both the main study and the sub-study at the same time, despite the fact that only a proportion of those taking part in the main study will be asked to take part in the sub-study. Consent will be sought for access to clinic data. Consent will also be sought for young people to be contacted before their follow-up assessment as a reminder and after this assessment if they have not attended. One copy of the signed consent form will be given to the participant, one for the clinical notes, and one will be kept by the study coordinator to file in the participant’s personal study register.

***Enrolment, randomisation (both conditions) and baseline assessments (standard-of-care condition only)***

The study coordinator will arrange the enrolment visit at the end of the consent process if the study coordinator has taken consent. If the clinician has taken consent, the study coordinator will contact the participant to arrange the enrolment visit. If the study coordinator and the participant are able to carry out the enrolment process at the same time as consent is obtained, this will be offered. At the enrolment visit, the participant will be enrolled into the study and given a study ID. Personal information will be recorded by the study coordinator in the participant’s personal study register (name, contact details, etc.) and non-identifiable information will be recorded in the central study register (study ID, date of enrolment, etc.).

Stratified block random allocation to condition by country/site (UK inner city NHS clinics and Uganda) will be used. There will be 12 blocks of either 15 or 16 participants per block (six blocks in Uganda, five blocks in London and one block in Birmingham). Computer-generated randomisation will be used using the Research Randomizer. The randomisation will be carried out by the study PI in the UK and the site PI in Uganda. Allocation will be concealed from the study coordinator in each country through the use of sequentially numbered opaque envelopes. The study coordinator will inform the participant which condition that they have been allocated to.

If the participant is in the standard-of-care condition, the baseline assessment will then be administered by the study coordinator, who will also record clinical information from the clinical notes or database for participants in this condition. Travel expenses will be paid to participants in both the intervention and the standard-of-care conditions if any have been incurred and a receipt will be kept.

***Baseline assessment (intervention condition) and intervention***

If the participant has been allocated to the intervention condition, their details will be passed to the intervention therapists, who will liaise with the study coordinator and participant about the timetabling of the baseline assessment and intervention sessions. The participant will be asked for their preference about when they would like intervention sessions to take place. The therapists will also explore any concerns that the participant has about the intervention. The baseline assessment will be administered by the study coordinator immediately prior to the first intervention session. The study coordinator will also record clinical information from the clinical notes or database.

All four intervention sessions will take place within a month time period. It is hoped that intervention participants will attend all four sessions, but they will be allowed to move on the next session if they have missed a previous one. All intervention sessions will be taped. Session attendance will be recorded by the therapists. A random selection of 10% of the sessions will be reviewed by one from the research team for fidelity to the intervention (using an intervention checklist), and feedback will be offered to the therapists based on this review (as well as problem-solving supervision). Travel expenses will be paid to participants by the therapists if any have been incurred and a receipt will be kept and passed on to the study coordinator. Participants will be remunerated for each intervention session.

***Post-intervention measurement***

This will take place for the intervention group only. It will take place at the end of the final session. This will be carried out by the study coordinator. Travel expenses will be paid if any have been incurred and participant payments made, and receipts will be kept by the study coordinator.

***Follow-up measurement***

This will occur 6 months after the baseline measures have been completed for both participants in the intervention condition and the standard-of-care condition and will be conducted by the study coordinator. We will use participant reminders and prompts (provided by the study coordinator) to minimise attrition. Participants will be contacted 1 month and 1 week before the second/third study visit is due (by phone/SMS), and attempts will be made to arrange the location and time of this visit. Participants will be reimbursed for their time and for any travel expenses incurred. Receipts will be kept by the study coordinator.

***After the follow-up assessment***

The following activities will take place after the follow-up assessment (by the study coordinator).

- Attempts to track participants if they have not attended their follow-up visit (by phone/SMS)
- Obtaining clinic data

**Phase 2: sub-study**

***Sampling and identification***

For the sub-study, participants in both conditions will be identified by the study coordinator to ensure that a range of characteristics are represented (e.g. age, location and sex) at each interview time point (post-intervention and follow-up).

***Approach***

The study coordinator will contact young people who have been identified to ask whether they would like to participate in the sub-study. If they are willing to participate, this will be recorded in their personal study register and the central study register. The study coordinator will arrange a time and location for the interview with the participant. If potential participants are not interested in taking part, this will be recorded in anonymised form (with the reason for refusal) in the central study register. This will help to determine the sub-study response rate.

***Participation***

The study coordinator will provide a reminder a week before the interview (by phone/SMS). Participants will be reimbursed for their involvement in the sub-study and for travel expenses incurred, and receipts will be kept by the study coordinator.

***After the arranged interview***

Attempts to track participants will be made by the study coordinator if they did not attend their interview visit (by phone/SMS).

### Data management and analysis

Confidentiality of all information will be assured to all participants unless they report something that suggests that they or someone else whom they identify might be at risk of serious harm. In this case, a member of the research team may need to speak to their clinical team so that they are able to provide appropriate support. All survey and interview data will be de-identified and treated as strictly confidential. Personally identifiable data will be stored anonymously in locked filing cabinets at each study site or digitally in an encrypted file. Data will be identified only by the participant’s study ID and will be password protected with only the project investigators having access.

#### Phase 1

The qualitative intervention development and intervention process evaluation will be informed by thematic analysis [86]. Individual and focus group interviews will be recorded and initial codes will be generated from the audio data. Themes will be developed and reviewed from the initial codes. These themes will then be defined and named. These findings will be used to inform the intervention sessions in phase 2.

#### Phase 2: main study

Exploration of data will be conducted via pooled analysis as well as analysing by country, although the former is necessarily preliminary given issues of statistical power. The evaluation will present descriptive data on recruitment and retention rates and acceptability. We anticipate a retention rate of 68% based on previous studies [44]. If the retention rate in the study in each country is 68%, the 95% confidence intervals of this rate for each country (*n* = 94) would be ± 9.5% (i.e. confidence interval width of 19%). We feel that this is an acceptable level of precision to inform the planning of a future RCT and will be sufficient to answer the study research questions. Criteria for moving to a full trial will be based on a range of criteria, including acceptability (from individual interviews and rating scales), demand (based on recruitment and retention rates), implementation, adaptation (across the two settings) and limited efficacy. Reasons for refusal to enrol in the study will be assessed and comparisons will be made between participants and both clinic and national data on the population to assess the likelihood of selection bias.

For secondary outcomes, descriptive analysis will be conducted on frequencies, percentages, central tendency and variability of data. Missing data will be imputed if appropriate. The distribution of missing data will be assessed with Little’s Missing Completely at Random Test [87] before using Expectation Maximisation to impute missing data if the proportion of missing data is small. For multi-item measures developed or adapted for this study, Principal Components Analysis (PCA) will first be carried out using both orthogonal (varimax) and oblique (oblimin) rotations. Scree plots will be examined to determine the number of factors to extract and analyses will be re-run specifying the number of factors. Items will be dropped from the scale if factor loadings are low or if they load on more than one factor. If any items are dropped, PCA will be re-run. Cronbach’s alpha will calculated for the final total scale and its subscales, and item-total correlations will be examined.

The effect of the intervention on secondary outcomes will be carried out separately for each country. Two tailed tests will be used. Exploration via multivariate analysis will also be carried out if there is sufficient data to support this approach. Sufficient data refers both to missing data (although we will impute data if this is minimal, e.g., <5%) and to the number of observations (e.g., if attrition results in a lack of statistical power to investigate multivariate relationships).

Independent *t* tests (where parametric assumptions were met) and chi-squared tests will be used to compare baseline characteristics of those retained in the study to those lost to follow up to examine whether there are any systematic differences between these groups. We will interpret our findings on secondary outcomes in light of this ‘lost to follow up’ analysis. We will aim to obtain follow-up data from all participants enrolled in the study regardless of the extent of involvement in intervention activities.

The extent to which participants in the intervention condition naturally use HIV disclosure ‘change talk’ (language indicating preparation for and commitment to disclosing their HIV status) in the final individual session will be calculated using the Motivational Interviewing Skills Code (MISC) 2.5 coding manual [88]. The relationship between the frequency of disclosure change talk and any HIV status sharing at the 6-month data point will be calculated using logistic regression analysis.

#### Phase 2: sub-study

The analysis of the qualitative data will be undertaken using thematic analysis [86]. Findings will be compared with the quantitative results to add further depth of understanding to the evaluation of the proposed intervention.

### Advisory groups

Two advisory groups will be formed (one in the UK and one in Uganda) to consult with on the progress of the study. The groups will involve key stakeholders, including young people living with PAH, partners, friends and family members, health care workers, health academics, and representatives from support organisations and policy making organisations. We will aim to recruit individuals with a spread of knowledge and expertise. The advisory group meetings will take place on four occasions over the life of the study—at the start of phase 1, prior to phase 2 recruitment, during phase 2 recruitment, and at the end of the study. The terms of reference of the groups will include the following:

- Providing advice on recruitment and retention
- Providing advice on the development of strategic partnerships, engaging stakeholders, and dissemination
- Providing a forum for discussion of key study issues
- Promoting the study
- Providing advice on intervention content

### Dissemination

The study protocol has been registered and is available on the ISRCNT registry (10.1186/ISRCTN31852047). The results will be disseminated to all participants, their friends and families, researchers, healthcare providers, support organisations, policy makers and the funding body through summary documents, presentations, through a study website (http://pc.rhul.ac.uk/sites/headsup/) and a full report. Training workshops and seminars will be conducted at the end of the study to disseminate and discuss findings; a video describing the findings briefly will be prepared, and a free webinar will be organised to allow questions to be asked about the research. The findings will also be submitted to peer-reviewed scientific journals and presented at conferences to reach researchers and healthcare professionals.

## Discussion

Young people with PAH face challenges to positive well-being, reducing the risk of onward HIV transmission and adhering consistently to ART medication. The sharing of one’s HIV status may assist in the process of coping with these challenges and, therefore, supporting young people with PAH with their decisions to share their status should be a priority. The HEADS-UP study is the first feasibility study to focus on HIV sharing among young adults living with PAH. The current investigation will build upon and extend the established evidence base supporting the potential benefits of increased sharing among young adults with PAH [29]. The study is unique in its inclusion of young people from both high-income/low-prevalence and low-income/high-prevalence contexts. This study will be the first to address important gaps in understandings of acceptable and feasible ways of delivering sharing support for young people with PAH. The findings will inform the actions needed to enhance HIV status sharing and improve young people with PAH’s satisfaction with their sharing decision-making.

### Potential future implications

Findings from this feasibility study may inform the management of HIV sharing for young people living with PAH and will inform the development of a full RCT, if the intervention is feasible and acceptable. Outcomes of this study will provide valuable information to guide development and implementation of HIV disclosure intervention policy and strategies. The study team, consisting of health professionals and researchers with substantial experience of working with young people with PAH in the UK and Uganda, is uniquely positioned to develop and disseminate such an intervention.

## Trial status

The trial began recruitment of participants for phase 1 in the UK in September 2019, and in Uganda in October 2019. We expect to recruit the full sample for phase 1 near the end of March 2020. Phase 2 recruitment will begin in August 2020 in the UK and in October 2020 in Uganda, and we anticipate reaching our recruitment target by July 2021.

## Data Availability

Anonymised electronic data from the study will be transferred to a data repository (eg. Figshare or RHUL Research Data Archive) at the end of the study. This will be freely accessible under Creative Commons CC BY licence and will be retained for 10 years.

## Abbreviations

PAH: Perinatally acquired HIV
ART: Antiretroviral therapy
U=U: Undetectable = Untransmittable
